# Supplementary material for: Molecular insights into intrinsic transducer-coupling bias in the CXCR4-CXCR7 system
Source: Nat Commun. 2023 Aug 9;14:4808. doi: 10.1038/s41467-023-40482-9 (PMC10412580; doi:10.1038/s41467-023-40482-9)

Supplementary Information

Molecular insights into intrinsic transducer-coupling bias in the CXCR4-CXCR7 system

Parishmita Sarma[1], Carlo Marion C. Carino[2], Deeksha S[1], Shubhi Pandey[1], Hemlata Dwivedi-Agnihotri[1], Xue Rui[3], Yubo Cao[4], Kouki Kawakami[2], Poonam Kumari[5], Yu-Chih Chen[6], Kathryn E. Luker[7], Prem N. Yadav[5], Gary D. Luker[7,8], Stéphane A. Laporte[5,9], Xin Chen[3], Asuka Inoue[2], and Arun K. Shukla[1*]

- **Supplementary Fig. 1**
- **Supplementary Fig. 2**
- **Supplementary Fig. 3**
- **Supplementary Fig. 4**
- **Supplementary Fig. 5**
- **Supplementary Fig. 6**
- **Supplementary Fig. 7**
- **Supplementary Fig. 8**
- **Supplementary Fig. 9**
- **Supplementary Table 1**
- **Source Data file :Supplementary Fig. 6a**
- **Source Data file :Supplementary Fig. 6c**

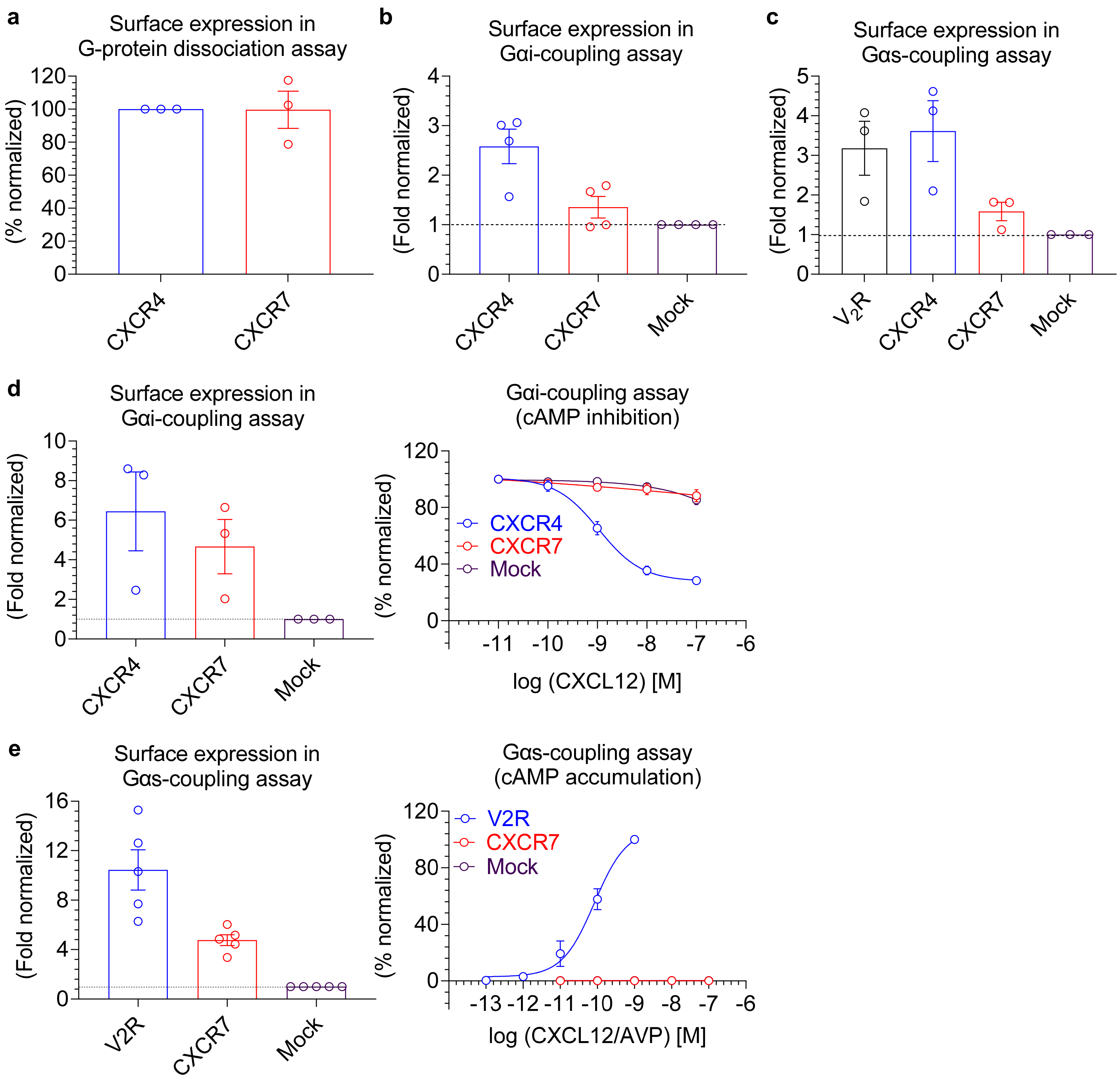

Supplementary Fig. 1. Surface expression of CXCR4 and CXCR7 in G-protein assays. a Surface expression of CXCR4 and CXCR7 in the G-protein dissociation assay measured using flow cytometry assay (mean±SEM; n=3 independent experiments; normalized with CXCR4 as 100%). **b-c** Surface expression of the indicated receptors in cAMP assay as measured using whole cell ELISA (mean±SEM; n=4 independent experiments for panel **b** and n=3 independent experiments for panel **c**; normalized as fold over mock (pcDNA)-transfection). **d-e** Surface expression (whole cell ELISA) and second messenger response (Gαi/s-coupling assay) under higher expression levels of CXCR4 and CXCR7 (mean±SEM; n=3 independent experiments; for panel **d** and n=5-6 independent experiments for panel **e**; for receptor surface expression: n=5 independent experiments, for Gαs-coupling assay; n=6) (Gαi/s-coupling assay in panel **d** and **e** are normalized with minimal ligand dose for CXCR4 and maximal signal for V_2R treated as 100%, respectively; surface expression data are normalized as fold over mock (pcDNA)-transfection). Dotted line in the plots b, c, d, and e indicates 1 fold. Source data are provided as a source data file.

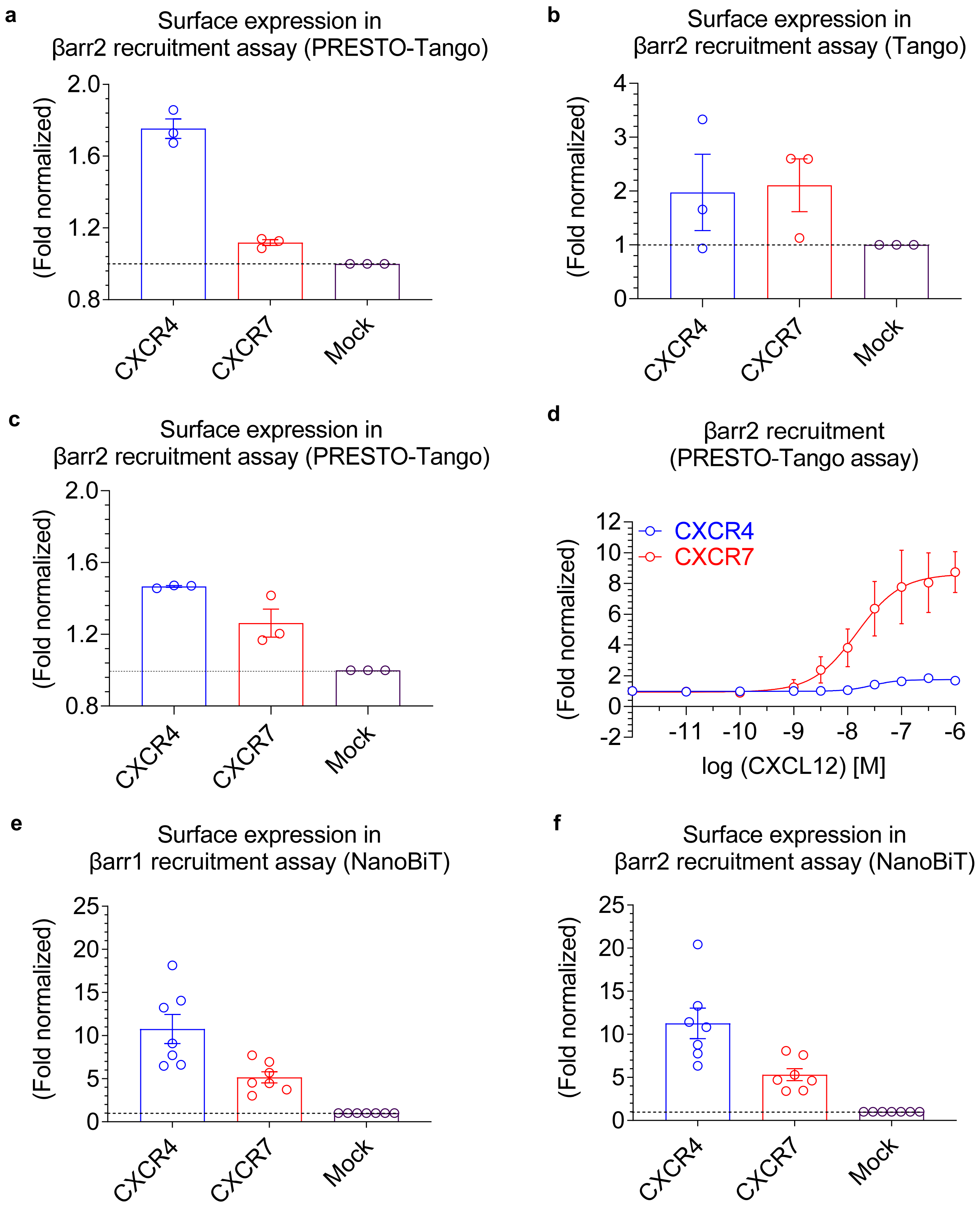

Supplementary Fig. 2. Surface expression of CXCR4 and CXCR7 in βarr recruitment assays. a-c, e-f Surface expression of CXCR4 and CXCR7 in βarr recruitment assays measured using whole cell ELISA (mean±SEM; n=3 independent experiments for panel a-b; n=7 independent experiments for panel e-f; n=3 independent experiments for panel c; normalized as fold over mock (pcDNA)-transfection). **d** CXCL12-induced βarr2 recruitment to CXCR4 and CXCR7 in PRESTO-Tango assay (mean±SEM; n=3 independent experiments; normalized with the luminescence signal at minimal ligand dose treated as 1). Dotted line in the plots a, b, c, e, and f indicates 1 fold. Source data are provided as a source data file.

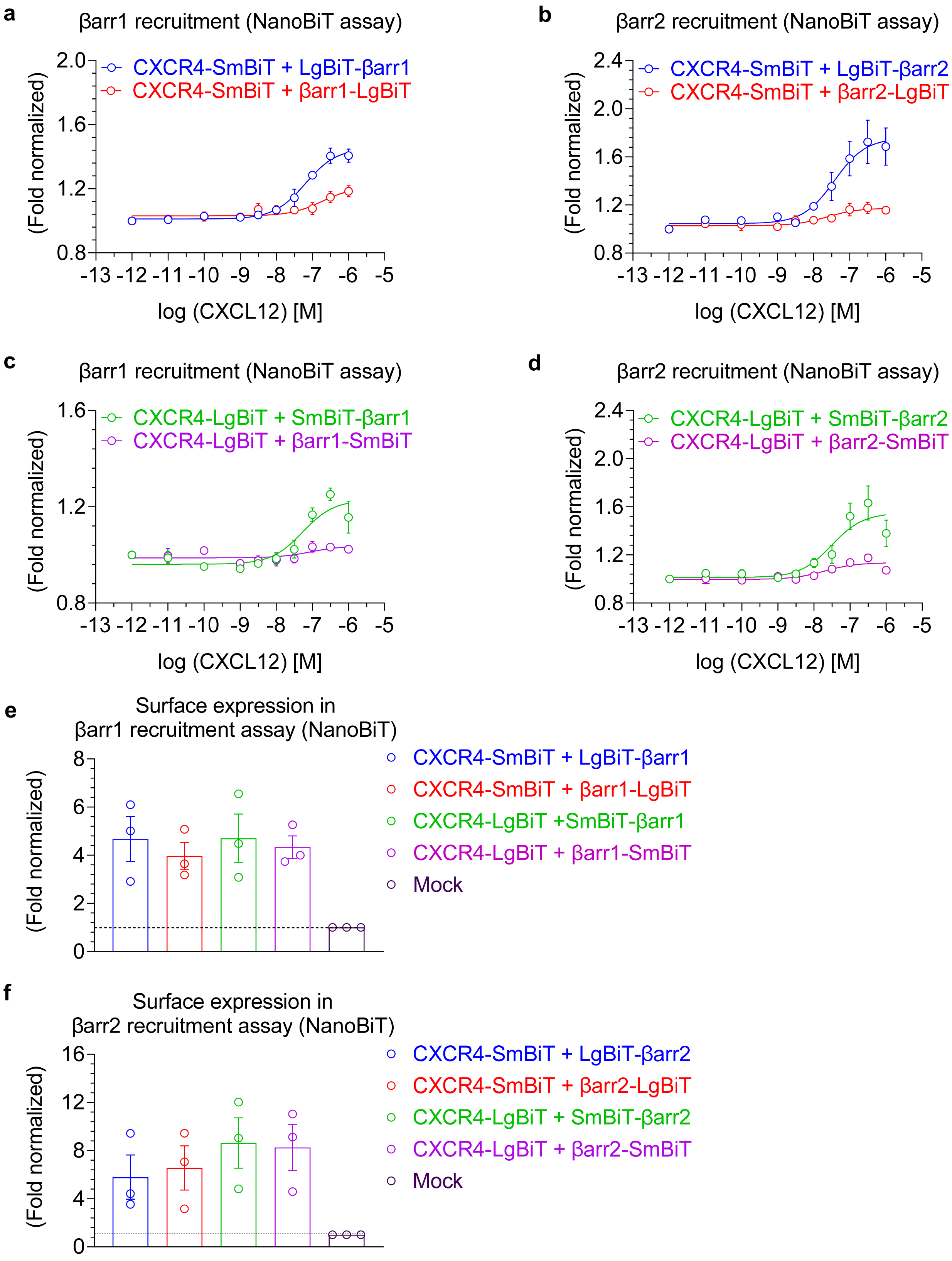

Supplementary Fig. 3. Optimization of NanoBiT-based βarr recruitment assay for CXCR4. a-d CXCL12-induced βarr1/2 recruitment to CXCR4 in NanoBiT assay with indicated combination of the constructs (mean±SEM; n=3 independent experiments; normalized with the luminescence signal at minimal ligand dose treated as 1). **e-f** Surface expression of CXCR4 constructs under indicated transfection conditions in the βarr recruitment assay as measured using whole cell ELISA (mean±SEM; n=3 independent experiments; normalized as fold over mock (pcDNA)-transfection). Dotted line in the plots e and f indicates 1 fold. Source data are provided as a source data file.

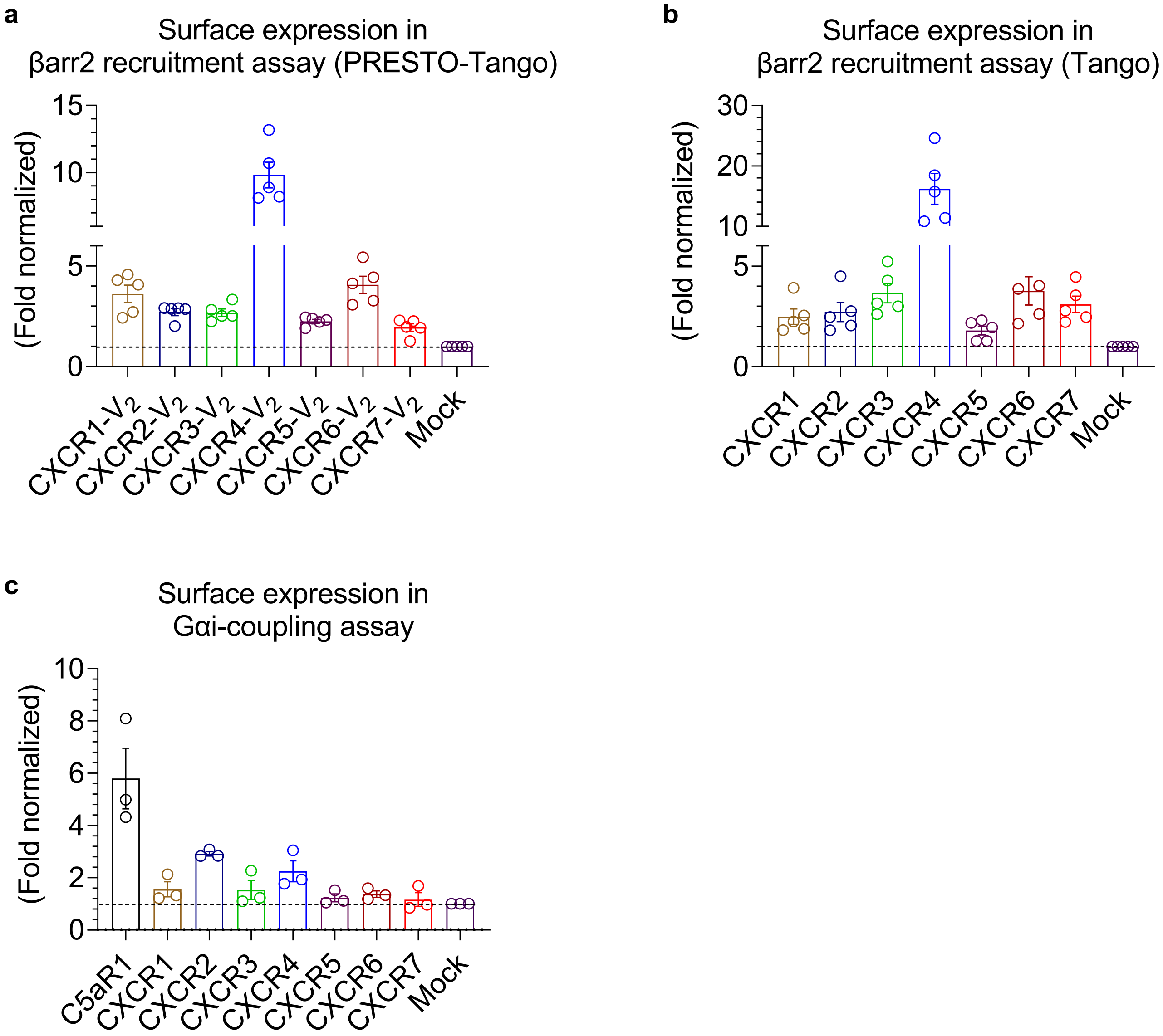

Supplementary Fig. 4. Surface expression of CXCRs. a-b Surface expression of indicated receptors in βarr recruitment assays measured using whole cell ELISA (mean±SEM; n=5 independent experiments for panel a-b; normalized as fold over mock(pcDNA)-transfection). **c** Surface expression of the indicated receptors in cAMP assay as measured using whole cell ELISA (mean±SEM; n=3 independent experiments; normalized as fold over mock (pcDNA)-transfection). Dotted line in plots a, b, and c indicates 1 fold. Source data are provided as a source data file.

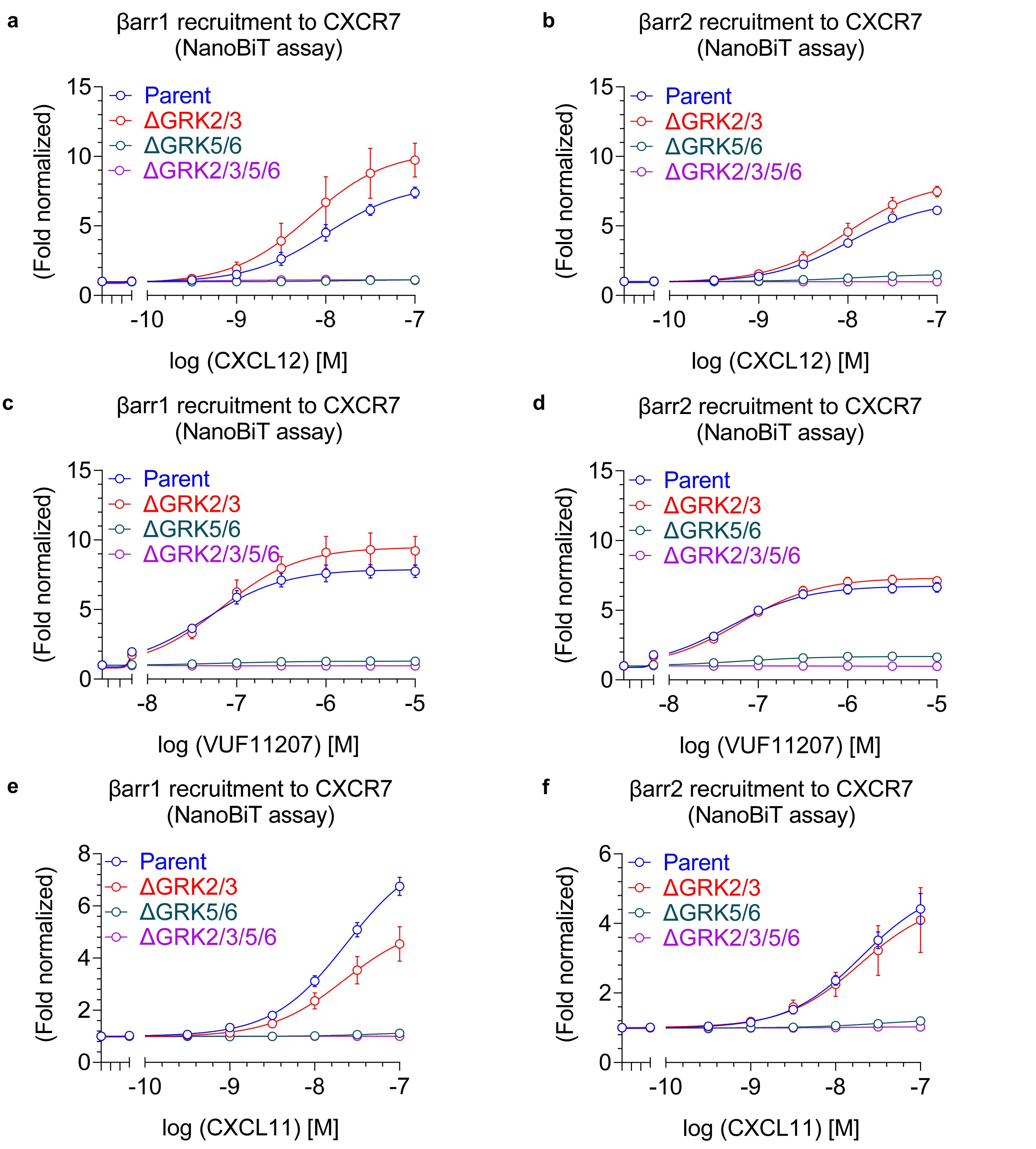

Supplementary Fig. 5. Effect of PTX treatment on βarr recruitment. a-f CXCL12-, VUF11207-, and CXCL11-induced βarr1 and 2 recruitment to CXCR7 in GRK knock-out cells using the NanoBiT assay with co-expression of the catalytic subunit of pertussis toxin (PTX) (Receptor-SmBiT+LgBiT-βarr1/2), respectively (mean±SEM; n=4-5 independent experiments; i.e., for CXCL12- and VUF11207-induced βarr1 recruitment: Parent, n=4; ΔGRK2/3, n=4; ΔGRK5/6, n=5; ΔGRK2/3/5/6, n=4; for CXCL12-induced βarr2 recruitment: Parent, n=5; ΔGRK2/3, n=4; ΔGRK5/6, n=4; ΔGRK2/3/5/6, n=4; for VUF11207-induced βarr2 recruitment: Parent, n=5; ΔGRK2/3, n=4; ΔGRK5/6, n=5; ΔGRK2/3/5/6, n=4; for CXCL11-induced βarr1 and βarr2 recruitment: Parent, ΔGRK2/3, ΔGRK5/6, ΔGRK2/3/5/6, n=4; normalized as fold response with respect to the unstimulated condition treated as 1). Source data are provided as a source data file.

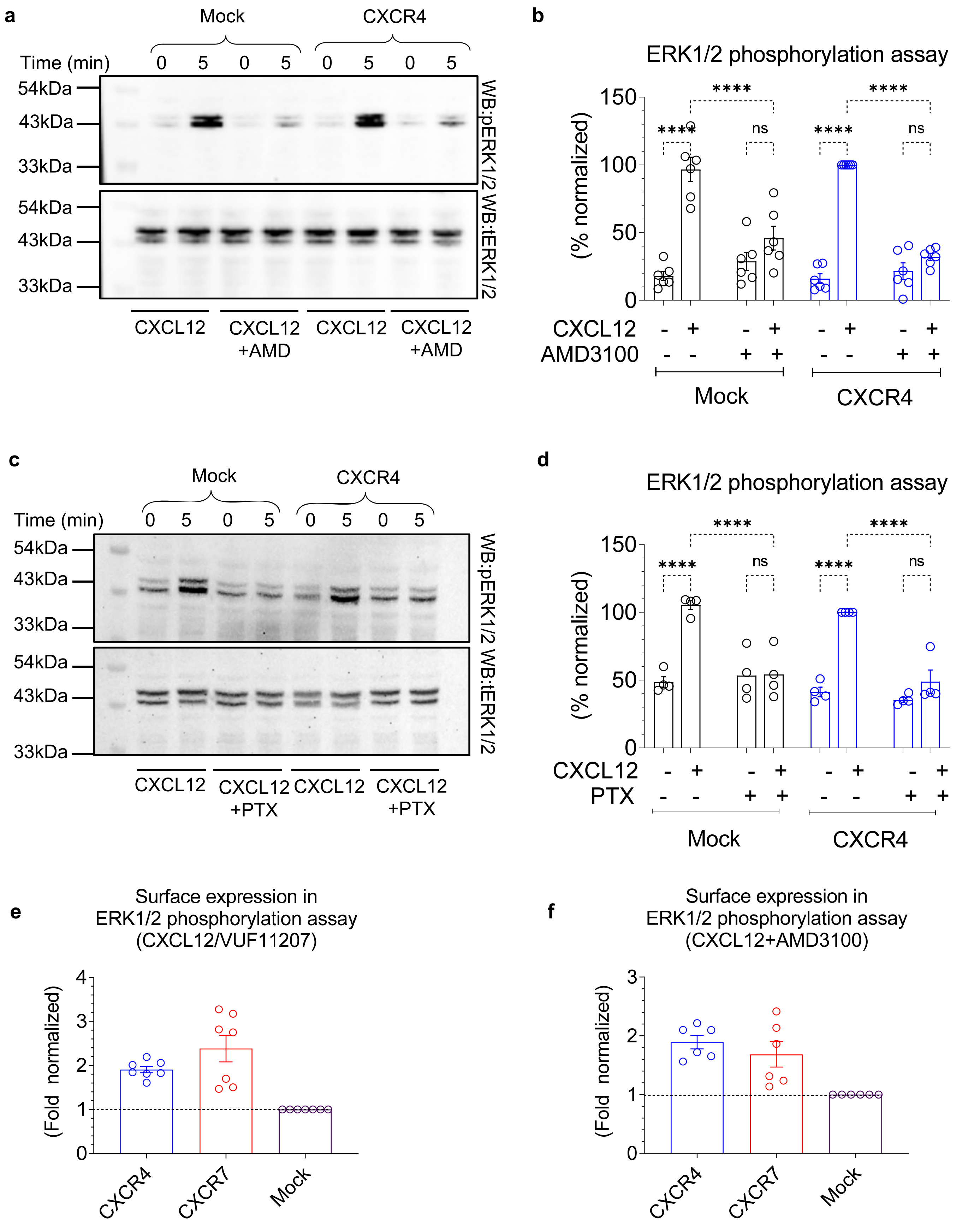
a
Mock
CXCR4
Time (min)
0 5 0 5 0 5 0 5
54kDa
43kDa
33kDa
WB:pERK1/2
WB:tERK1/2
CXCL12
CXCL12 +AMD
CXCL12
CXCL12 +AMD
b
ERK1/2 phosphorylation assay
(% normalized)
150
100
50
0
****
****
****
****
ns
ns
CXCL12
AMD3100
Mock
CXCR4
c
Mock
CXCR4
Time (min)
0 5 0 5 0 5 0 5
54kDa
43kDa
33kDa
WB:pERK1/2
WB:tERK1/2
CXCL12
CXCL12 +PTX
CXCL12
CXCL12 +PTX
d
ERK1/2 phosphorylation assay
(% normalized)
CXCL12
PTX
Mock
CXCR4
e
Surface expression in ERK1/2 phosphorylation assay (CXCL12/VUF11207)
(Fold normalized)
4
3
2
1
0
CXCR4
CXCR7
Mock
f
Surface expression in ERK1/2 phosphorylation assay (CXCL12+AMD3100)
(Fold normalized)
3
2
1
0
CXCR4
CXCR7
Mock

Supplementary Fig. 6. Effect of AMD3100 and PTX treatment on ERK1/2 phosphorylation. a-d CXC12-induced ERK1/2 phosphorylation in HEK-239 cells transfected with either pcDNA (mock) or CXCR4 plasmid with and without pre-treatment of AMD3100 and PTX as indicated. Panels b and d present densitometry-based quantification of data presented in panels a and c (mean±SEM; n=6 and n=4 independent experiments, respectively; normalized with 5 min stimulation of CXCR4 condition without AMD3100/PTX; Two-way ANOVA, Tukey's multiple comparison test. The exact *p* values for panel b are as follows: mock-AMD3100: 0 min vs. mock-AMD3100: 5 min (p < 0.0001), mock-AMD3100: 5 min vs. mock+AMD3100: 5 min (*p* < 0.0001), mock+AMD3100: 0 min vs. mock+AMD3100: 5 min (*p* = 0.4441), CXCR4-AMD3100: 0 min vs. CXCR4-AMD3100: 5 min (*p* < 0.0001), CXCR4-AMD3100: 5 min vs. CXCR4+AMD3100: 5 min (*p* < 0.0001), CXCR4+AMD3100: 0 min vs. CXCR4+AMD3100: 5 min (*p* = 0.8975). The exact *p* values for panel d are as follows: mock-PTX: 0 min vs. mock-PTX: 5 min (*p* < 0.0001), mock-PTX: 5 min vs. mock+PTX: 5 min (*p* < 0.0001), mock+PTX: 0 min vs. mock+PTX: 5 min (*p* > 0.9999), CXCR4-PTX: 0 min vs. CXCR4-PTX: 5 min (*p* < 0.0001), CXCR4-PTX: 5 min vs. CXCR4+PTX: 5 min (*p* < 0.0001), CXCR4+PTX: 0 min vs. CXCR4+PTX: 5 min (*p* = 0.7397) (*****p* < 0.0001, ns= non-significant). **e-f** Surface expression of CXCR4 and CXCR7 in ERK1/2 phosphorylation assay was measured using whole cell ELISA (mean±SEM; n=7 independent experiments for panel e and n=6 independent experiments for panel f, normalized as fold-over mock (pcDNA)-transfection. Dotted line in the plots e and f indicates 1 fold. Source data are provided as a source data file.

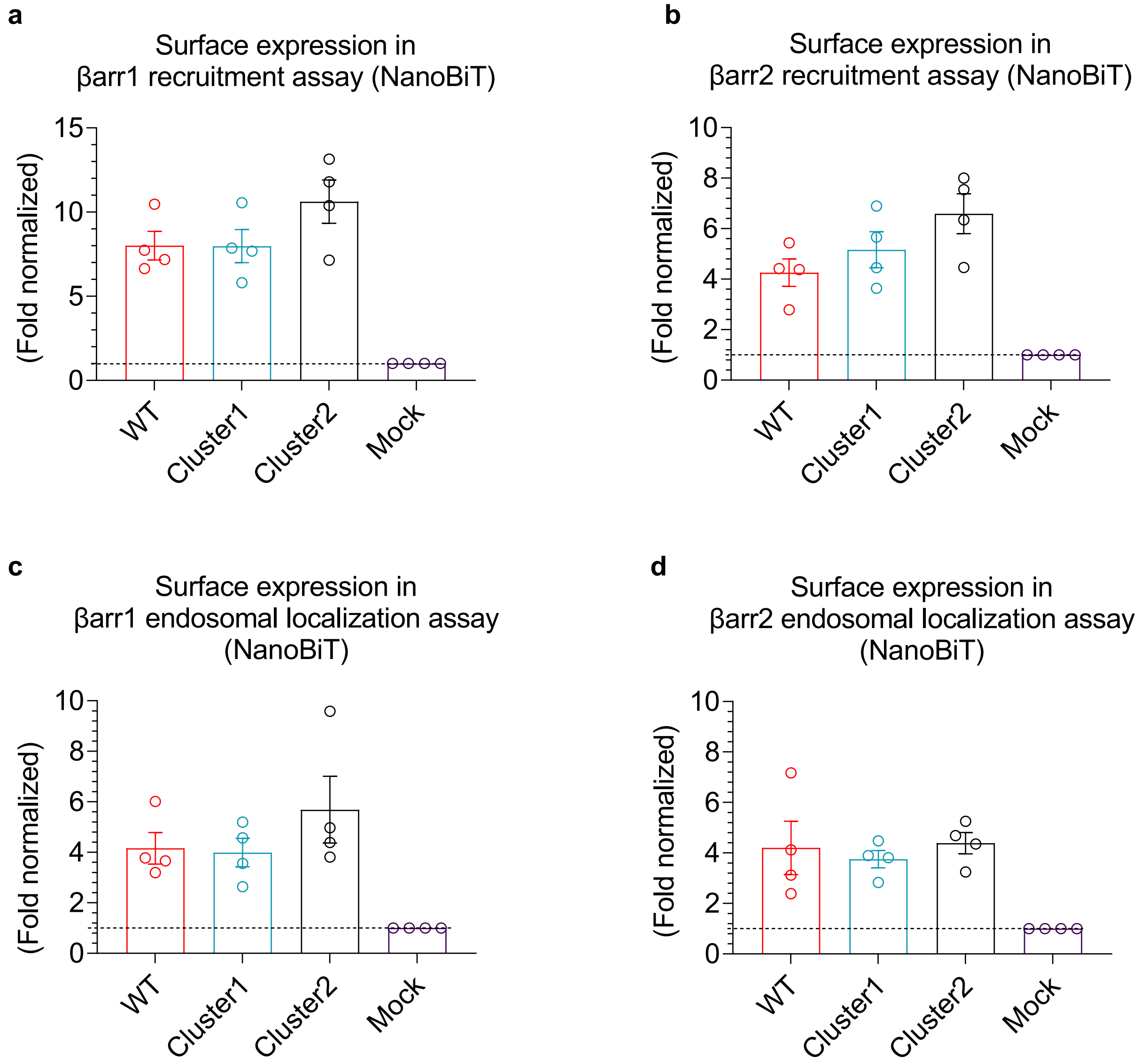

Supplementary Fig. 7. Surface expression of CXCR7 mutants in different assays. a-e Surface expression of indicated receptors was measured in different assays using whole cell ELISA (mean±SEM; n=4 independent experiments; normalized as fold over mock (pcDNA)-transfection). Dotted line in the plots a, b, c, and d indicates 1 fold. Source data are provided as a source data file.

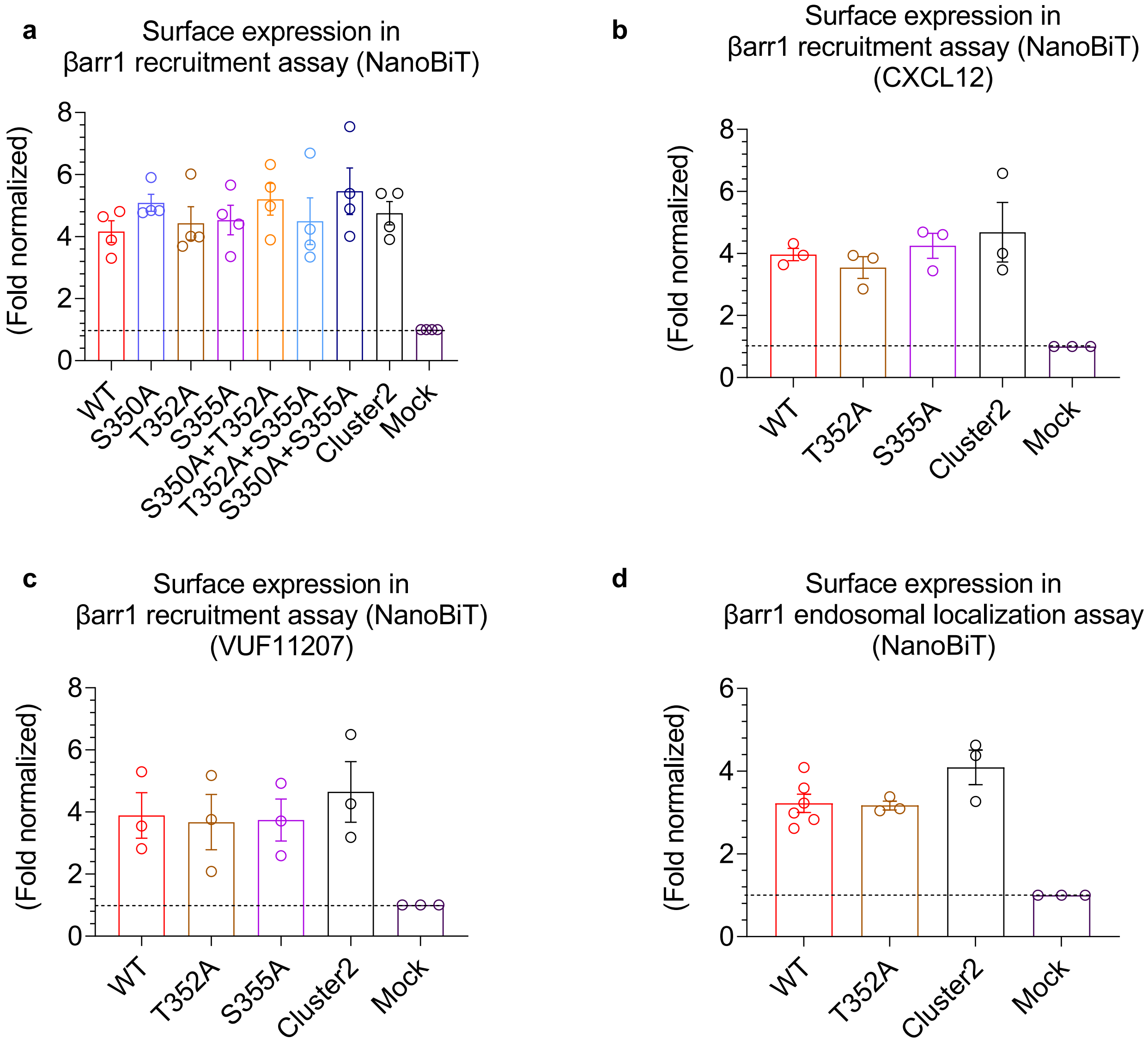

Supplementary Fig. 8. Surface expression of CXCR7 mutants in different assays. a-d Surface expression of indicated receptors were measured in different assays using whole cell ELISA (mean±SEM; n=4 independent experiments for panel a, n=3 independent experiments for panel b and c, n=3-6 independent experiments for panel d; for βarr1 endosomal localization: WT, n=6; T352A, n=3; and Cluster2, n=3 normalized as fold over mock (pcDNA)-transfection). Dotted line in the plots a, b, c, and d indicates 1 fold. Source data are provided as a source data file.

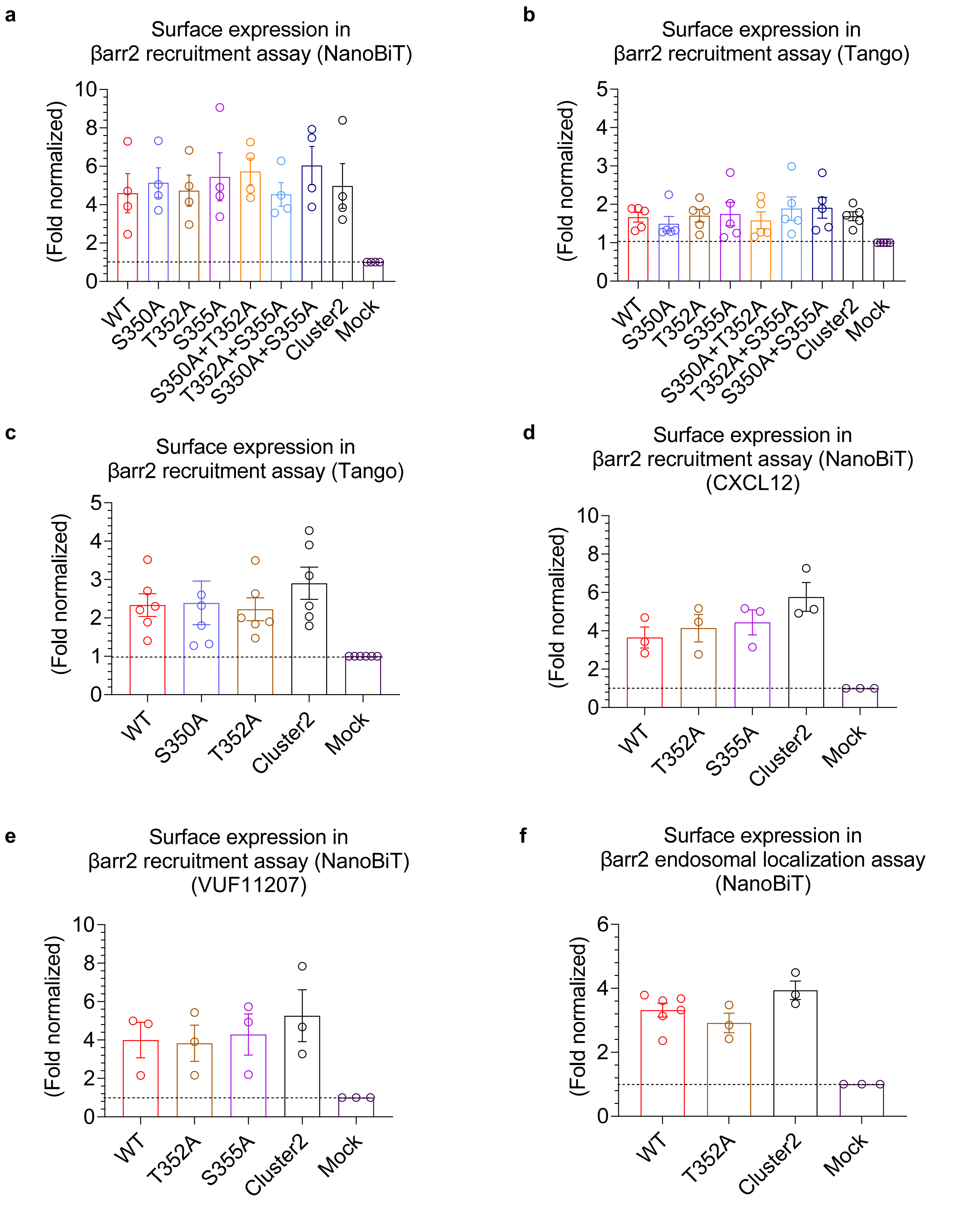

Supplementary Fig. 9. Surface expression of CXCR7 mutants in different assays. a-f Surface expression of indicated receptors were measured in different assays using whole cell ELISA (mean±SEM; n=4 independent experiments for panel a, n=5 for panel b, n=6 for panel c, n=3 for panel d and e, n=3-6 for panel f; βarr2 endosomal localization: WT, n=6; T352A, n=3; and Cluster2, n=3; normalized as fold over mock (pcDNA)-transfection). Dotted line in the plots a,b,c,d, and e indicates 1 fold. Source data are provided as a source data file.

Supplementary Table 1. List of the primers used in the study.		
**Construct**	**Primer**	**Sequence**
CXCR1 Tango	CXCR1_Fw	CGCGGATCC ATGTCCAATATCACCG
	CXCR1_Rv	CCGGAATTC CAAATTTGAAGAGACATTAAC
CXCR2 Tango	CXCR2_Fw	CGCGGATCCATGGAGGACTTCAACATGGAG
	CXCR2_Rv	CCGGAATTCCAGGGTCGTGGAGGTG
CXCR3 Tango	CXCR3_Fw	CGCGGATCCATGGTTCTGGAAGTTTCC
	CXCR3_Rv	CCGGAATTCGAGGCCAGAATAACTAGC
CXCR4 Tango	CXCR4_Fw	CGCGGATCCATGGAGGGGATATCAATC
	CXCR4_Rv	CCGGAATTCAGAGCTATGAAATGAGCTG
CXCR5 Tango	CXCR5_Fw	CGCGGATCCATGAACTATCCCCTGACC
	CXCR5_Rv	CCGGAATTCAAAGGTGGTCAGGCTAG
CXCR6 Tango	CXCR6_Fw	CGCGGATCCATGGCCGAACACGAC
	CXCR6_Rv	CCGGAATTCCAGCTGGAACATGGAGG
CXCR7(S350A)	CXCR7_S350A_Fw	CTCCAGAGTCGCCGAGACGGAGTAC
	CXCR7_S350A_Rv	GCATCGATGAGCTTGGTG
CXCR7(T352A)	CXCR7_T352A_Fw	AGTCTCAGAGGCCGAGTACTCTGCC
	CXCR7_T352A_Rv	CTGGAGGCATCGATGAGC
CXCR7(S355A)	CXCR7_T355A_Fw	GACGGAGTACGCCGCCTTGGAGC
	CXCR7_T355A_Rv	TCTGAGACTCTGGAGGCAT
CXCR7(S350A+T352A)	CXCR7_S350A+T352A_Fw	AGTCGCCGAGGCCGAGTACTCTG
	CXCR7_S350A+T352A_Rv	CTGGAGGCATCGATGAGC
CXCR7(T352A+S355A)	CXCR7_T352A+S355A_Fw	GGCCGAGTACGCCGCCTTGGAGC
	CXCR7_T352A+S355A_Rv	TCTGAGACTCTGGAGGCATC
CXCR7(S350A+S355A)	CXCR7_S350A+S355A_Fw	GACGGAGTACGCCGCCTTGGAGCAG
	CXCR7_S350A+S355A_Rv	TCGGCGACTCTGGAGGCA
CXCR7_Cluster1	CXCR7_Cluster1_Fw	AGGGCTCGCCAAGCTCATCGATGCCTCC
	CXCR7_Cluster1_Rv	GCTTTGGCCGCGTACTTGAAGATGAAGGCC
CXCR7_Cluster2	CXCR7_Cluster2_Fw	GAGTACGCTGCCTTGGAGCAGAGCACC
	CXCR7_Cluster2_Rv	CGCCTCTGCGACTCTGGAGGCATCGATG
CXCR7_cSmBiT	CXCR7_cSmBiT_Fw	CGGGGTACCGAGGAGATCTGCCACCATGGGC
	CXCR7_cSmBiT_Rv	TCCCCCGGGTTTGGTGCTCTGCTCCAAG
CXCR4_cSmBiT	CXCR4_cSmBiT_Fw	CGGGGTACCGAGGAGATCTGCCACCATGGGC
	CXCR4_cSmBiT_Rv	TCCCCCGGGAGAGCTATGAAATGAGCTGG
CXCR4_LgBiT	CXCR4_LgBiT_Fw	CGGGGTACCGAGGAGATCTGCCACCATGGGC
	CXCR7_LgBiT_Rv	TCCCCCGGGAGAGCTATGAAATGAGCTGG

Source Data file :Supplementary Fig. 6a

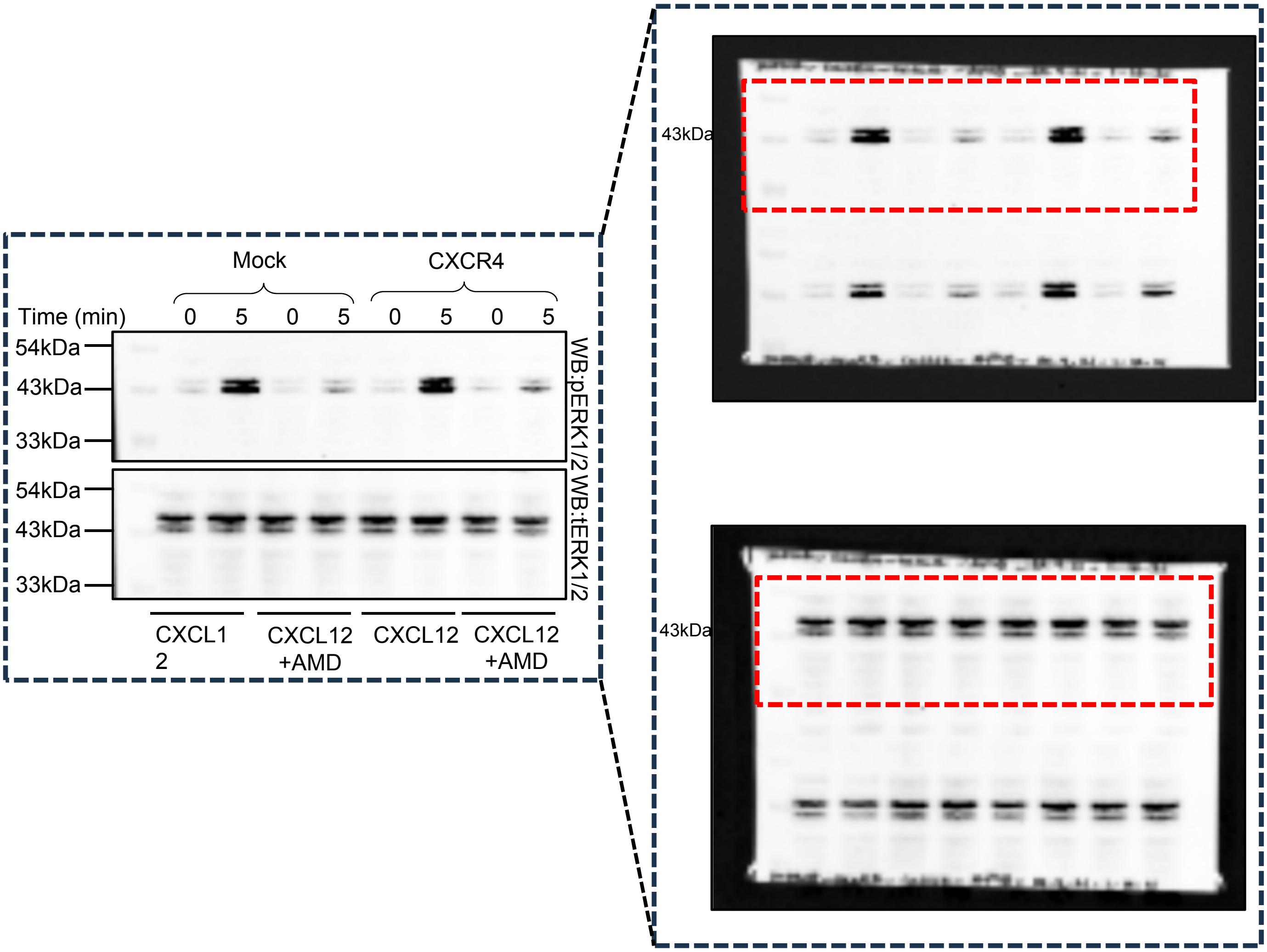

Source Data file :Supplementary Fig. 6c

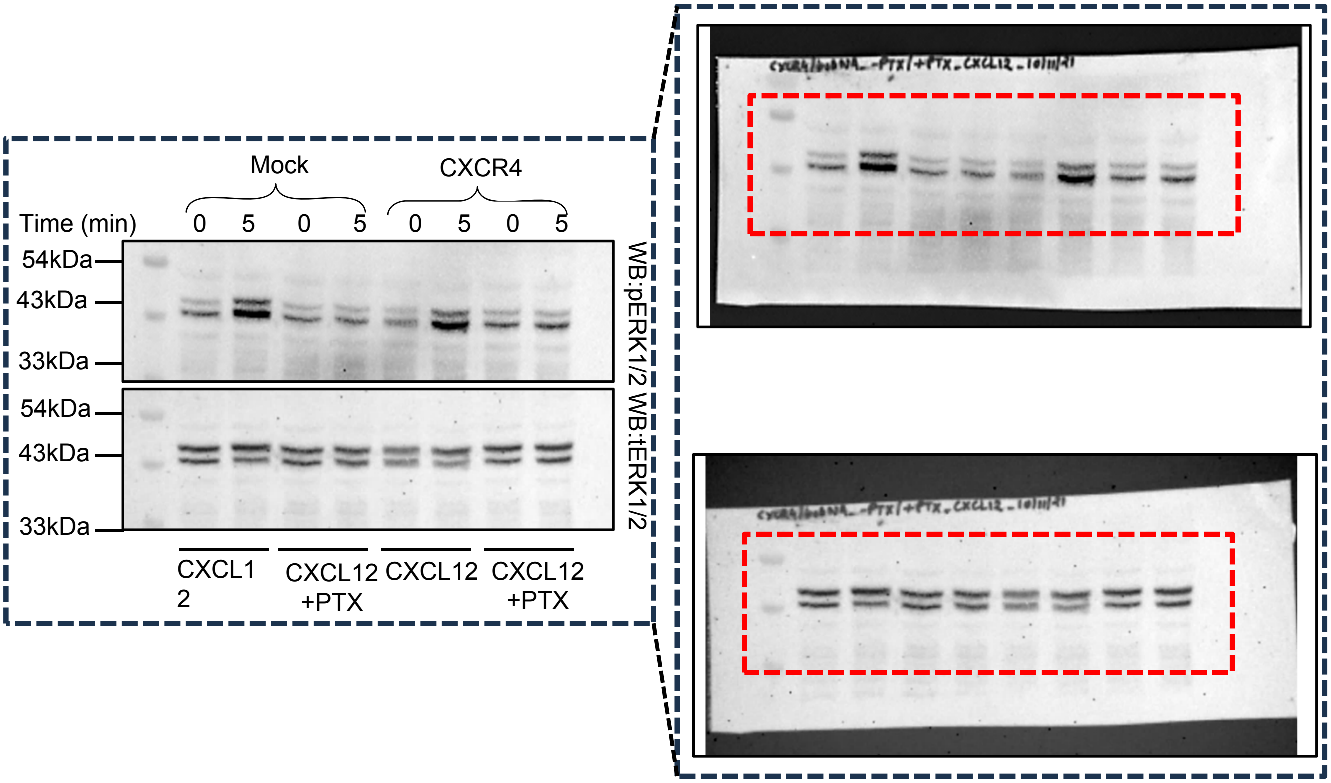

Supplement: Supplementary file 1 — Supplementary Information [file 41467_2023_40482_MOESM1_ESM.pdf]
