# Supplementary material for: Molecular insights into intrinsic transducer-coupling bias in the CXCR4-CXCR7 system
Source: Nat Commun. 2023 Aug 9;14:4808. doi: 10.1038/s41467-023-40482-9 (PMC10412580; doi:10.1038/s41467-023-40482-9)
Supplement: Supplementary file 3 — Reporting Summary [file 41467_2023_40482_MOESM3_ESM.pdf]

nature portfolio

Corresponding author(s): Arun K. Shukla

Last updated by author(s): 31-07-2023

Reporting Summary

Nature Portfolio wishes to improve the reproducibility of the work that we publish. This form provides structure for consistency and transparency in reporting. For further information on Nature Portfolio policies, see our Editorial Policies and the Editorial Policy Checklist.

Statistics

For all statistical analyses, confirm that the following items are present in the figure legend, table legend, main text, or Methods section.

n/a	Confirmed	
☐	☒	The exact sample size (*n*) for each experimental group/condition, given as a discrete number and unit of measurement
☐	☒	A statement on whether measurements were taken from distinct samples or whether the same sample was measured repeatedly
☐	☒	The statistical test(s) used AND whether they are one- or two-sided<br>*Only common tests should be described solely by name; describe more complex techniques in the Methods section.*
☒	☐	A description of all covariates tested
☐	☒	A description of any assumptions or corrections, such as tests of normality and adjustment for multiple comparisons
☐	☒	A full description of the statistical parameters including central tendency (e.g. means) or other basic estimates (e.g. regression coefficient) AND variation (e.g. standard deviation) or associated estimates of uncertainty (e.g. confidence intervals)
☐	☒	For null hypothesis testing, the test statistic (e.g. *F*, *t*, *r*) with confidence intervals, effect sizes, degrees of freedom and *P* value noted<br>*Give P values as exact values whenever suitable.*
☒	☐	For Bayesian analysis, information on the choice of priors and Markov chain Monte Carlo settings
☒	☐	For hierarchical and complex designs, identification of the appropriate level for tests and full reporting of outcomes
☒	☐	Estimates of effect sizes (e.g. Cohen's *d*, Pearson's *r*), indicating how they were calculated

Our web collection on statistics for biologists contains articles on many of the points above.

Software and code

Policy information about availability of computer code

Data collection	Omega series microplate plate reader, BMG LABTECH, VictorX4 Perkinelmer plate reader, Bio-rad ChemiDoc Imaging System, imageQunatLAS 500, SpectraMax L, molecular devices, Flex Station 3 molecular devices,
Data analysis	Following tools and softwares were used for data analysis:<br><br>Biochemical and cellular assays: Image Lab software, Bio-rad ChemiDoc Imaging System, FlowJo software<br><br>For Cellular data analysis and graph plot: GraphPad Prism-9.5.0

For manuscripts utilizing custom algorithms or software that are central to the research but not yet described in published literature, software must be made available to editors and reviewers. We strongly encourage code deposition in a community repository (e.g. GitHub). See the Nature Portfolio guidelines for submitting code & software for further information.

Data

Policy information about availability of data

All manuscripts must include a data availability statement. This statement should provide the following information, where applicable:

- Accession codes, unique identifiers, or web links for publicly available datasets
- A description of any restrictions on data availability
- For clinical datasets or third party data, please ensure that the statement adheres to our policy

Data generated and presented in the study is available in the provided source data file and supplementary information files.
All other data are available from the corresponding authors upon reasonable request.

Research involving human participants, their data, or biological material

Policy information about studies with human participants or human data. See also policy information about sex, gender (identity/presentation), and sexual orientation and race, ethnicity and racism.

Reporting on sex and gender	No human research participants were included in this study.
Reporting on race, ethnicity, or other socially relevant groupings	No human research participants were included in this study.
Population characteristics	No human research participants were included in this study.
Recruitment	No human research participants were included in this study.
Ethics oversight	No human research participants were included in this study.

Note that full information on the approval of the study protocol must also be provided in the manuscript.

Field-specific reporting

Please select the one below that is the best fit for your research. If you are not sure, read the appropriate sections before making your selection.

☒ Life sciences ☐ Behavioural & social sciences ☐ Ecological, evolutionary & environmental sciences

For a reference copy of the document with all sections, see nature.com/documents/nr-reporting-summary-flat.pdf

Life sciences study design

All studies must disclose on these points even when the disclosure is negative.

Sample size	As appropriate for different functional assays, we carried out at least three independent biological replicates for each experiment (exact number of replicates is mentioned in the corresponding figure legend). No predetermined statistical method was used to choose minimal sample size, based on the sample variability and heterogeneity sample size were choosen. The sample sizes selected or use in the study were sufficient to define the observed result.
Data exclusions	No data were excluded from the analysis.
Replication	All reported results are replicable. For each experiments, we performed multiple biological replicates. Details of the number of biological replicates are mentioned in the corresponding figure legends.
Randomization	No sample randomization was applied. Since this is not a animal or clinical case study, randomization was neither needed not attempted .
Blinding	No blinding was attempted or required as all the data used in the study. Blinding was not necessary or possible for cell based or functional assays because in most cases data was collected and analyzed by individual investigator.

Reporting for specific materials, systems and methods

We require information from authors about some types of materials, experimental systems and methods used in many studies. Here, indicate whether each material, system or method listed is relevant to your study. If you are not sure if a list item applies to your research, read the appropriate section before selecting a response.

Materials & experimental systems

n/a	Involved in the study
☐	☒ Antibodies
☐	☒ Eukaryotic cell lines
☒	☐ Palaeontology and archaeology
☒	☐ Animals and other organisms
☒	☐ Clinical data
☒	☐ Dual use research of concern
☒	☐ Plants

Methods

n/a	Involved in the study
☒	☐ ChIP-seq
☐	☒ Flow cytometry
☒	☐ MRI-based neuroimaging

Antibodies

Antibodies used	1.Monoclonal Anti-FLAG® M2-Peroxidase (HRP) antibody: Supplier: Sigma-Aldrich, #Cat no. A8592.<br>2.Phospho-p44/42 MAPK (Erk1/2) (Thr202/Tyr204) Antibody, Supplier: Cell signaling technology (CST), #Cat no. 9101<br>3.p44/42 MAPK (Erk1/2) (Thr202/Tyr204) Antibody, Supplier: Cell signaling technology (CST), #Cat no. 9102<br>4.Anti-rabbit IgG secondary antibody, Suppler genescript #cat no. A00098<br>5.Anti-DYKDDDDK monoclonal antibody, supplier: FujiFilm Wako Chemicals # cat no. 012-22384<br>6.Alexa Fluor 488-conjugated goat anti-mouse igG secondary antibody, supplier: Thermo Fisher Scientific #cat no. A11001
Validation	1.Monoclonal Anti-FLAG® M2-Peroxidase (HRP) antibody is commercially available and validated by the manufacturer for use .https://www.sigmaaldrich.com/IN/en/product/sigma/a8592<br>2.https://www.cellsignal.com/products/primary-antibodies/phospho-p44-42-mapk-erk1-2-thr202-tyr204-antibody/9101<br>3.https://media.cellsignal.com/s3sds/9102-sds-AGHS-EN-20171127130322000.pdf<br>4.https://www.genscript.com/antibody/A00098-Goat_Anti_Rabbit_IgG_Antibody_H_L_HRP_pAb<br>5.https://labchem-wako.fujifilm.com/us/product/detail/W01W0101-2238.html<br>6.https://www.thermofisher.com/antibody/product/Goat-anti-Mouse-IgG-H-L-Cross-Adsorbed-Secondary-Antibody-Polyclonal/A-11001

Eukaryotic cell lines

Policy information about cell lines and Sex and Gender in Research

Cell line source(s)	HEK-293 cells were obtained from ATCC (cat. no. CRL-3216), and thermo fisher scientific (cat no. R70507)<br>MDA-MB-231 were purchased from ATCC, Manassus, VA, USA
Authentication	None of the cell lines used were authenticated.
Mycoplasma contamination	Cell lines were not tested for mycoplasma contamination.
Commonly misidentified lines<br>(See ICLAC register)	No commonly misidentified cell lines were used.

Flow Cytometry

Plots

Confirm that:

☒ The axis labels state the marker and fluorochrome used (e.g. CD4-FITC).

☒ The axis scales are clearly visible. Include numbers along axes only for bottom left plot of group (a 'group' is an analysis of identical markers).

☒ All plots are contour plots with outliers or pseudocolor plots.

☒ A numerical value for number of cells or percentage (with statistics) is provided.

Methodology

Sample preparation	HEK-293 cells were transfected with FLAG-epitope-tagged receptor and seeded in 96 well plate flowed by labeling with the anti-DYKDDDDK monoclonal antibody (10 µg/ml), followed by the Alexa Fluor 488-conjugated secondary antibody (10 µg/ml).
Instrument	EC800 Flow cytometer (Sony)
Software	FlowJo software
Cell population abundance	N/A (since we analyzed single cell type and obtained data from all observed cells).

Gating strategy	N/A (since we used all of the recorded fluorescence signal and calculated mean value, description of gating strategy or the plot representation was not performed).

☒ Tick this box to confirm that a figure exemplifying the gating strategy is provided in the Supplementary Information.
