# Supplementary material for: Molecular insights into intrinsic transducer-coupling bias in the CXCR4-CXCR7 system
Source: Nat Commun. 2023 Aug 9;14:4808. doi: 10.1038/s41467-023-40482-9 (PMC10412580; doi:10.1038/s41467-023-40482-9)
Supplement: Supplementary file 4 — Source Data [file 41467_2023_40482_MOESM4_ESM.zip › Source_Data_Blots.pptx]

## Slide 1
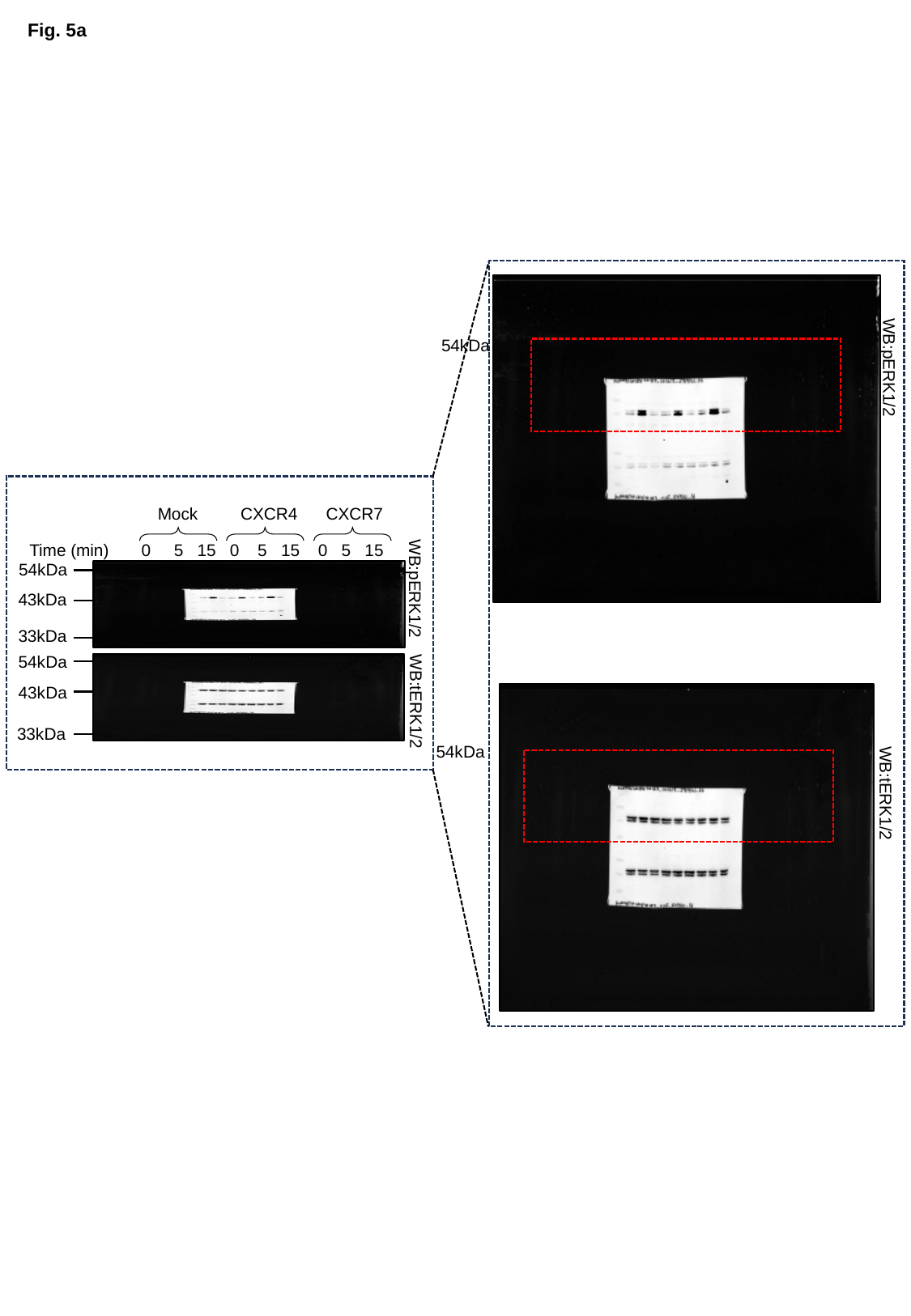

Fig. 5a
WB:pERK1/2
54kDa
CXCR7
Mock
CXCR4
CXCR7
Mock
CXCR4
Time (min) 0 5 15 0 5 15 0 5 15
WB:pERK1/2
WB:tERK1/2
54kDa
43kDa
33kDa
54kDa
43kDa
33kDa
54kDa
WB:tERK1/2

## Slide 2
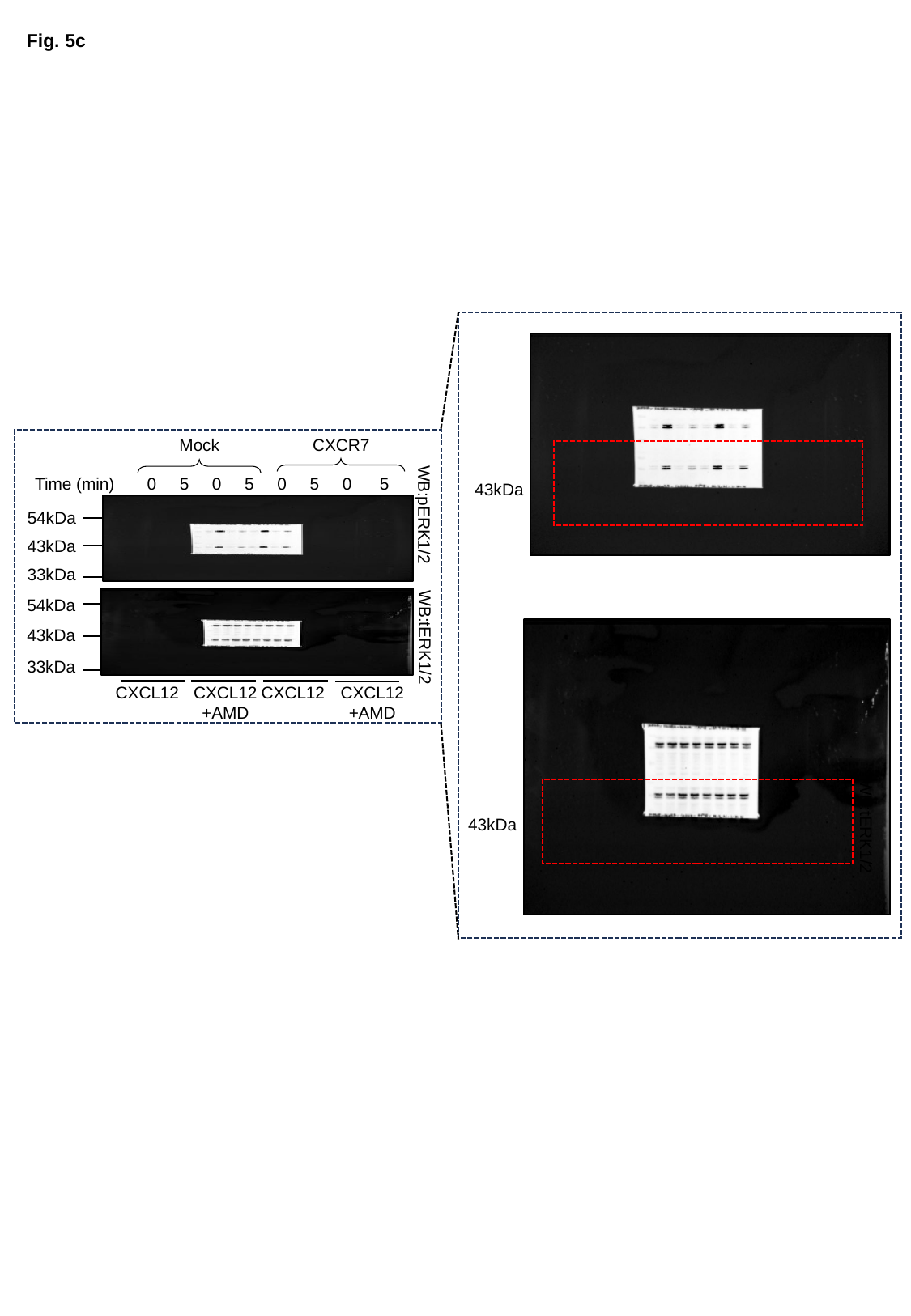

Fig. 5c
WB:pERK1/2
43kDa
Mock
CXCR7
Time (min) 0 5 0 5 0 5 0 5
54kDa
43kDa
33kDa
54kDa
43kDa
33kDa
WB:tERK1/2
WB:pERK1/2
CXCL12
CXCL12
+AMD
CXCL12
CXCL12
+AMD
43kDa
WB:tERK1/2

## Slide 3
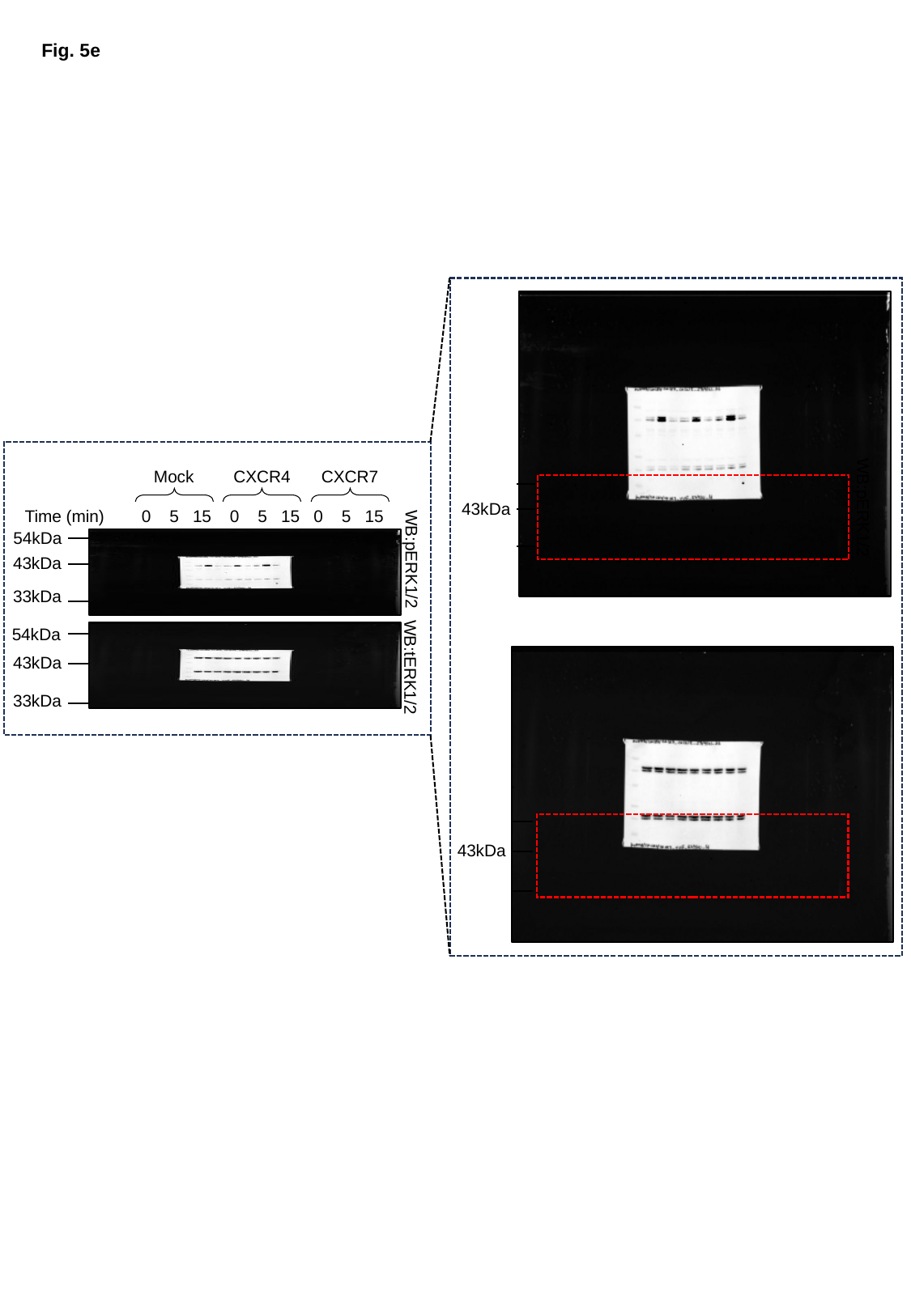

Fig. 5e
43kDa
WB:pERK1/2
Mock
CXCR4
CXCR7
Time (min) 0 5 15 0 5 15 0 5 15
54kDa
43kDa
WB:pERK1/2
33kDa
43kDa
33kDa
WB:tERK1/2
54kDa
43kDa
WB:tERK1/2
